# The Effect of the β-Al_5_FeSi Phases on Microstructure, Mechanical and Fatigue Properties in A356.0 Cast Alloys with Higher Fe Content without Additional Alloying of Mn

**DOI:** 10.3390/ma14081943

**Published:** 2021-04-13

**Authors:** Lenka Kuchariková, Denisa Medvecká, Eva Tillová, Juraj Belan, Michaela Kritikos, Mária Chalupová, Milan Uhríčik

**Affiliations:** 1 Department of Materials Engineering, Faculty of Mechanical Engineering, University of Žilina, Univerzitná 8215/1, 010 01 Žilina, Slovakia; denisa.medvecka@fstroj.uniza.sk (D.M.); eva.tillova@fstroj.uniza.sk (E.T.); juraj.belan@fstroj.uniza.sk (J.B.); maria.chalupova@fstroj.uniza.sk (M.C.); milan.uhricik@fstroj.uniza.sk (M.U.); 2 Faculty of Material Science and Technology in Trnava, Slovak University of Technology in Bratislava, J. Bottu 25, 917 24 Trnava, Slovakia; michaela.kritikos@stuba.sk

**Keywords:** β-Al_5_FeSi intermetallic phases, higher content of Fe, dismissing Mn addition, properties of aluminum alloys with higher Fe content

## Abstract

Secondary-cast aluminum alloys have increasing industrial applications. Their biggest deficiency is their impurity content, especially Fe, which has low solubility in Al and almost all the content creates intermetallic phases. This work examines the effect of higher Fe content on the microstructure and properties of A356.0 alloy. At the same time, no other possibility existed to affecting the brittleness of the formation of the β phases. The calculation of Fe_crit_, ratio of Mn/Fe, quantitative and computed tomography analysis of porosity and Fe plate-like phases, measurement of mechanical and fatigue properties, and fractography analysis were performed in this study. The results show that gravity die casting into a sand mold, and the non-usage of Mn addition or heat treatment, do not have a negative effect on increasing the size of the Fe-rich plate-like phases. The longest Fe-rich phases have limited the pore growth and ratios, but their higher thickness led to greater porosity formation. The mechanical and fatigue properties correlate with the Fe_crit_ level and the highest were for the experimental alloy with 0.454 wt.% of Fe. The experimental results confirmed the fact that if the Fe plate-like phases have a length of up to 50 µm, the fatigue properties depend more on the size of porosity. If the length of the Fe needles is more than 50 µm, then the properties are mainly affected by the length of these Fe phases.

## 1. Introduction

In terms of production, Aluminum alloys take second place in the world after iron-based alloys. The general need for weight-saving to reduce fuel consumption leads increasing interest in aluminum alloys for automotive applications. It should be noted that the production of a ton of primary aluminum requires an order of magnitude higher energy consumption compared to the production of a ton of iron-based alloy. Therefore, it is very urgent to comprehensively increase the use of the secondary aluminum alloys (made from scrap metal processing), the production of which allows a reduction of energy consumption by up to twenty times (secondary only requires about 2.8 kWh/kg to produce, while primary requires about 45 kWh/kg), compared to primary production, with a significantly lower environmental load [1,2,3]. The recycling of aluminum scrap can save not only energy, but other raw materials, as well [4].

Casts from secondary alloys have a higher content of Fe than those from primary alloys. This is related to the recycling process, since the removal of Fe is costly and difficult [5,6]. A higher Fe content can lead to the formation of a higher quantity of unwanted brittle platelet Fe-rich intermetallic phases with sharp edges, categorized as plate-like, which causes premature fracture in castings [7,8,9]. Therefore, the presence of Fe, even in small amounts, degrades the mechanical properties of aluminum alloys such as strength, fatigue, fracture toughness, and especially ductility, since it forms the β-Al_5_FeSi intermetallic phase in a plate-like morphology (needle-like in 2D cross-section) [7,9,10,11]. There are two main α Fe-rich phases formed be Al–Si alloys: α-Al_15_(Mn, Fe)_3_Si_2_ (Chinese script-type structure with a cubic (bcc) crystal lattice) and α-Al_8_Fe_2_Si (phase is hexagonally shaped). The other, β, phases, identified in Al-Si alloys, are Al_3_FeSi_2_, Al_4_FeSi, Al_9_Fe_2_Si_2_, Al_5_FeSi, Al_4.5_FeSi, and Al_5_Fe_2_Si [9,11,12,13,14]. Additions of Mn, Cr, Cu, V, Mo, and W promote a body-centered cubic structure in the Fe-rich phase. For instance, when Mn is added, as the proportion of Mn to Fe increases, the precipitation of the Fe-rich phase may take a series of forms from hexagonal (Al_8_Fe_2_Si), to bcc (Al_15_(FeMn)_3_Si_2_), to the simple cubic (Al_15_Mn_3_Si_2_) [11,15,16]. The commonly accepted Mn content needed to promote α-Fe (Chinese script or skeleton-like appearance) depends on the Fe content and the calculated Fe/Mn ratio, which should be below 2:1 (Mn/Fe = 0.5). The addition of Mn is chosen when the critical content of Fe exceeds 0.45 [8,11,17].

The concentration of internal stress fields over the intermetallic phases causes sudden rupture and complete failure of the studied materials [10,18]. The most critical aluminum cast components are subjected to cyclic loading conditions [19]. Therefore, the main requirements in the final products made from aluminum alloys are sufficient strength-to-weight ratio, good fatigue properties, good formability or castability, high corrosion resistance, recyclability, and low production expenditures [20,21,22]. Several material defects, including porosity, oxide films, and intermetallic inclusions may facilitate fatigue crack initiation, reduce product lifetime, and decrease cyclic strength, especially at a high number of cycles [23]. Therefore, a deep understanding of microstructure and defect formation during the solidification process is important to produce reliable castings for fatigue-critical applications [24]. Hence it is necessary to investigate the effect of the Fe-rich intermetallic phases and porosity on the evolution of fatigue damage.

This work was focused on the experimental investigation of changes to the microstructure, mechanical properties, and fatigue life of secondary cast aluminum alloys of the AlSi_7_Mg_0.3_ type, with emphasis on the application of recycling materials, which leads to the presence of a higher amount of Fe, affecting the formation of a higher content of Fe plate-like phases and porosity.

## 2. Materials and Methods

Eutectic and near eutectic Al–Si alloys are widely used in the casting industry due to their superior abrasion and corrosion resistance, low thermal expansion coefficient, and high strength/weight ratio [5,22]. The A356.0 (AlSi_7_Mg_0.3_) cast alloy belongs to the Al–Si alloys commonly used for applications such as cylinder blocks, cylinder heads, and pistons. These applications require the best properties [18]. The increasing usage of secondary alloys for casting production comes with changes to their microstructure and properties. Fe is a common and undesirable impurity in aluminum alloys. Primary aluminum alloys typically contain between 0.02 and 0.15 wt.% of Fe, commonly in average of ~0.07–0.10 wt.%. The Fe content depends on the quality of the incoming ore and control of the various processing parameters and other raw materials. Secondary aluminum alloys typically contain between 0.25–0.8 wt.% of Fe, commonly in average of 0.4–0.7 wt.% for alloys cast under high pressure [17,25].

The company UNEKO, Ltd., Zátor, Czech Republic wants to increase the usage of secondary alloys cast in sand molds. Therefore, AlSi_7_Mg_0.3_ cast alloys were used for experimental analyses with the main intention being to investigate the higher content of Fe without additional alloying with Mn or heat treatment, for the influence of the formation of brittle Fe-based intermetallic phases in the plate-like (needle) form, as well as their effect on the alloy’s microstructure, mechanical and fatigue properties. To examine the effect of higher Fe content, the melt was designedly contaminated (Table 1). The contamination was chosen with respect to the common Fe content in secondary alloys (0.4–0.7 wt.%), although the EN 1706 standard [26] indicates a permitted value of up to 1.4 wt.% of Fe for alloys cast into a metallic mold under high pressure. The chemical composition of the primary AlSi_7_Mg_0.3_ experimental alloy (designated A) was obtained from the standard (EN 1706). Alloys with a higher Fe content were used as reference alloys with the designations B and C for the following experimental procedure (Table 1). The chemical composition of those alloys was determined by optical emission spectroscopy (OES).

The designed contamination of the melt took place at 750 ± 5 °C by adding an AlFe_10_ master alloy. Gravity die casting into a sand mold was used for the casting of the experimental materials. Materials were prepared in the form of rods with dimensions of 300 mm in length and 20 mm in diameter. The melt was not modified or refined. The addition of Mn and heat treatment were not used intentionally due to our prediction of how the higher content of Fe would affect the microstructure and properties of the experimental alloys. For such reasons, the critical Fe—levels were calculated according to Equation (1) and the Mn/Fe ratio [17]:

(1) $${\mathrm{Fe}}_{\mathrm{crit}}\approx 0.075\times \left(\%\mathrm{Si}\right)-0.05.$$

The raw experimental material, was cast into cavity-shaped sand molds, which were chosen because if the solidification/cooling rates were very high (e.g., high pressure die casting), supercritical iron contents may not have been detrimental, but as the cooling rate decreases (gravity die casting → sand casting, etc.), the probability of supercritical iron levels causing problems would dramatically increase [27].

**Figure 1:**
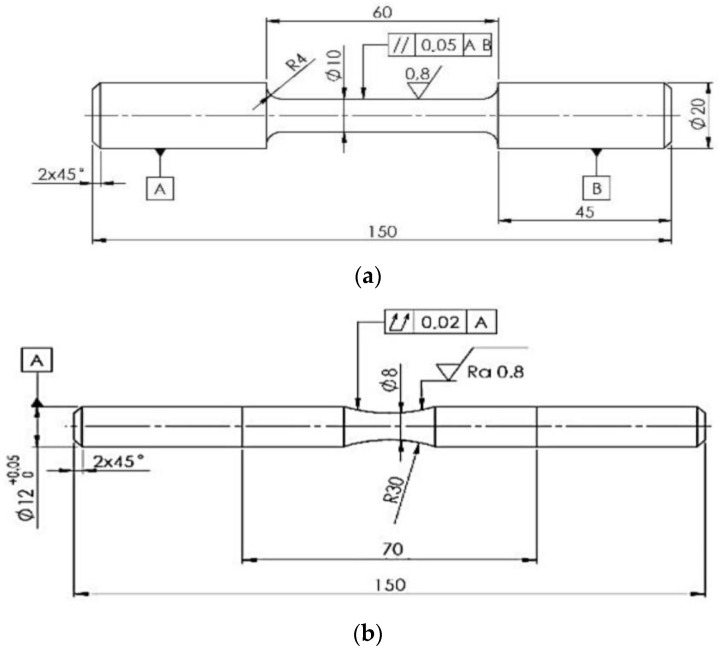
Specimens for the (**a**) mechanical and (**b**) fatigue tests.

The specimens for the mechanical and fatigue testing were made from the rods by means of a turning operation (Figure 1). Tensile properties (ultimate tensile strength (UTS), yield strength (YS), and reduction area (A)) were measured on an INSTRON Model 5985, INSTRON, Darmstadt, Germany, according to the ISO 6892-1:2009 standard [28]. The test rates and control were set according to the Method A recommended ranges. The hardness test was performed by using Brinell hardness testing (HBW) with a load of 2451.7 N, the testing ball diameter of 5 mm, and a dwell time of 15 s. The samples for the test were wet, ground, and then polished using 3 μm diamond paste. The mechanical characteristics obtained reflect the average values of at least six separate measurements for each experimental material. All experimental tests were performed at ambient temperature (22 ± 1 °C).

Cylindrical fatigue specimens (Figure 1b) were used for fatigue tests. The total length of the specimen was 150 mm, with an active zone diameter of ø8 mm. The tests were performed on an experimental device for rotation–bending fatigue testing. The fatigue life of the experimental alloys was obtained with different strain rates with a loading frequency f = 37.5 Hz, a load ratio of R = −1, and at room temperature T = 22 ± 1 °C. The recording of the number of applied cycles was determined using the speed counter. The fatigue life of the experimental alloys was tested on 12 test specimens from different production series (alloys A, B and C). The fatigue investigation of the cast aluminum alloys mainly concentrated on the regime below approx. 10^7^–10^8^ cycles to failure. More recent investigations in a very high cycle regime show that the fatigue properties at 10^9^ cycles cannot be accurately predicted, and that the lifetime may be either under- or overestimated when extrapolating from fatigue data obtained at fewer than 10^7^ or 10^8^ cycles [23]. Therefore, the fatigue tests were performed in the high-cycle fatigue area up to 10^7^ and the run-out condition was set to 1 × 10^7^ cycles. The fatigue life was established for 3 × 10^6^ cycles according to the demands of the UNEKO company. Fatigue life was evaluated by using S–N curves. Fractography analysis of the fatigue surfaces was performed using the scanning electron microscopy (SEM) on VEGA LMU II equipment.

**Figure 2:**
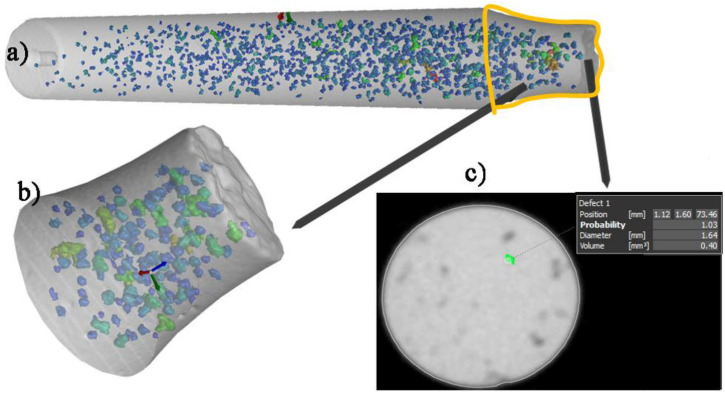
CT measurements of porosity on (**a**) specimens, (**b**) active zone of specimens and (**c**) the fracture surface.

The Fe-rich intermetallic phases and porosity formation in the experimental material were observed and documented by using light microscopy (the Carl ZEISS Jena NEOPHOT 32, Jena, Germany), and scanning electron microscopy (the TESCAN VEGA LMU II, Brno, Czech Republic), then linked to the energy-dispersive X-ray spectroscopy (the TESCAN Bruker’s QUANTAX EDX analyzer, Brno, Czech Republic). The metallography samples were selected from the casting rods used to creating the fatigue test specimens, which were prepared for metallography observation according to standard procedure (etched by HF). Some samples were also deeply etched for 30 s in HCl solution to reveal the 3D morphology of the presented phases. Quantitative metallography was carried out on the NIKON NIS Elements 4.2 image analyzer software, Prague, Czech Republic to quantify the amount (surface ratio), surface size of pores, and length of the Fe-rich phases (needles in the metallography samples). To minimize the statistical error in the calculation, more than 30 micrographs for each specimen were assessed, and the relative error was less than 0.05%. The porosity volume and volume ratio of the fatigue specimens were measured using computed tomography analysis on a CT Scan the Carl ZEISS METROTOM, Bratislava, Slovakia (Figure 2). The length of the Fe-rich needles on the fatigue specimens was measured to compare the results to those of the metallography assessment.

Since, in general, the company does not have reliable non-destructive techniques for detecting defects inside castings, the estimation of the presence of inclusions (porosity, in this study) was achieved using statistical analysis. Therefore, this study presents the usage of the largest extreme value distribution (LEVD) for porosity prediction [29,30,31]. The pore size measurement of the metallography samples was used as the unique measure of pore severity for the fatigue and pore comparison. In this method, a number *n* of separate control areas (measurements), each of the same area *S*_0_ of the selected image magnification of the polished surface prepared for quantitative analysis were chosen, and the parameter √*A* (area ½) of the largest pore within each region was recorded. The result was a set of *n* observations of maximum √*A*, or *x*_1_ through *x_n_*, and Gumbel extreme value distribution was fitted to these data.

The Gumbel distribution has the following distribution function [29,30,31]:

(2) G (x: λ, δ) = exp (−exp(−(x−λ)/δ)), (x ∈ R),

where δ measures variability > 0 and λ represents the largest pore’s typical size. In fact, λ is the *e*^−1^ quantile of distribution *G*, and is referred to as the characteristic largest inclusion size in area *A*_0_. Several methods are available for estimating the parameters λ and δ. The simplest method is a graphical procedure based on the Gumbel plot. In the graphical procedure, the data are the first ordered from smallest to largest as *x*_1_ ≤ *x*_2_ ≤ • • ≤ *x_n_*. Then, the Gumbel plot of −ln (−ln (j/(n + 1))) against x_j_ is drawn. Under the Gumbel distribution, the points are expected to lie close to the line with slope 1/δ and intercept −λ/δ. Estimates of these parameters may be obtained by *l* the least-square fitting a straight line through the data points [29,30,31]:

y = −ln (−ln (j/(n + 1))) = b_0_ + b_1_ (A)^1/2^. (3)

The relation between the distribution parameters and the regression coefficients is (4) [29,30,31]:

δ = 1/b_1_; λ = −b_0_/b_1_. (4)

The fitted distribution may be used to estimate the size of the largest inclusion in a plane region *S* larger than the control area *S*_0_, or to compare the largest inclusions from control areas of different sizes [29,30,31].

## 3. Results and Discussion

The Fe and Mn content control show that each experimental alloy had a Fe_crit_ exceeding 0.45 (Table 2). Taylor [17] stated that in aluminum alloys with 5 wt.% of Si, the critical iron content is ~0.35; at 7 wt.% of Si, it rises to ~0.5; at 9 wt.% of Si it is ~0.6, and at 11 wt.% of Si, it reaches ~0.75. In this case, Mn had to be added to alloys B and C, since the critical Fe content exceeded a value of 0.5 (but the difference between alloys A and B was only about 4.7% and for alloy C it was about 4.5%). Mn addition was intentionally unused, despite the fact that the Mn/Fe ratio did not reach a value of 0.5 (Table 2). Therefore, there was an assumption of greater formations of Fe-rich phases in platelet-like forms in experimental alloys, especially in alloy B (Table 2).

The results of the quantitative analysis gathered using the NIKON NIS Elements 4.2 image analyzer software, Prague, Czech Republic (the length of Fe-rich plate-like phases and the surface area and ratio of pores, Table 3) confirm the common knowledge about limiting the size of pores. Ceschini et al. [19] and Samuel et al. [32,33] show that although the branching of the β-platelets led to porosity formation, the platelets, on the other hand, also limit pore growth, which is confirmed by the results found in Table 3. An argument presented by Moustafa [34], that long-branched β-platelets result in the formation of large shrinkage cavities due to the inability of liquid metal to feed the space between them during solidification, and that the percentage of porosity, maximum pore area, and maximum pore length increases with an increase in the average maximum β-Al_5_FeSi platelet length [33], were not confirmed by the quantitative analysis of pore surface size (Table 3), because only the percentage of porosity (pore surface ratio) increased. This result could also be connected to the fact that the maximum length of the Fe plate-like phases (Table 3) in experimental alloys meets the common size range of 50–500 µm [25]. It could also be stated that the length of Fe-rich plate-like phases correlates with the Mn/Fe ratio and Fe_crit_ results because the highest length values were observed in samples from alloy B (Table 2 and Table 3). From the quantitative metallography results, it can be assumed that when the Fe levels exceed a value of ~0.5 (the content of Fe required to ensure the castability and machinability of secondary aluminum alloys) the percentage of porosity, maximum pore area and maximum pore length increase (the results for experimental alloy C) (Table 3).

**Figure 3:**
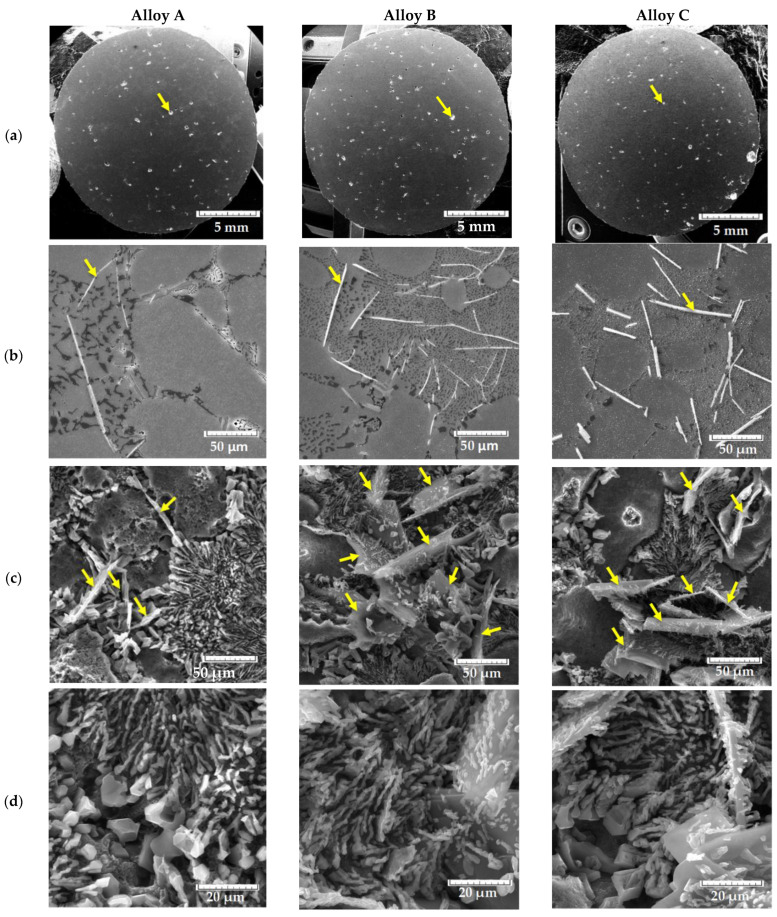
Formations of (**a**) porosity, (**b**,**c**) Fe plate-like phases, (**d**) eutectic Si in the experimental alloys.

The higher content of Fe in alloy C led to the formation of a shorter (Table 3) and thicker (Figure 3) needle phase in comparison to alloy B (Figure 3). These results correlate with the findings of Mathew [12], who determined that materials with 2 wt.% of Fe have thicker phases than materials with 0.6 wt.% of Fe.

The maximum length of the Fe plate-like phases in experimental alloy A (cast to sand mold, e.g., slow cooling rate) did not exceed the maximum size, as the primary alloys cast with very high cooling rates (from 10 to 50 µm [25]) did.

Alloy B had the largest length of the Fe plate-like phases, but they still did not reach 500 µm. The common knowledge that the iron-bearing intermetallics (especially β-Al_5_FeSi platelets and α-Al_15_(Fe, Mn)_3_Si_2_ script) can grow up to several millimeters in size in slowly cooled Al–Si alloy castings with high Fe and/or Mn levels [25] was also not confirmed (Table 3, Figure 3).

The assessment of the metallography samples only confirmed that the absence of Mn additions led to greater formation of the Fe plate-like phases (Figure 3). The formation of Fe-rich phases in plate-like form was also studied by using deep etching for the assessment of their 3D morphology (Figure 3c). On the metallography samples, β-Fe has the shape of needles (Figure 3b) and is therefore at times incorrectly called iron needles. Many works show that the compact and difficult shape of such phases has a detrimental effect on mechanical properties [8,12,25,35,36]. The β phases were observed in the form of long plates (Figure 3). In alloy A, these plates were mostly deployed separately (Figure 3c) in microstructure, but in alloys B and C, these phases were connected with each other as show in the results in the literature [25,35,36]. Such morphology leads to increased formation of porosity, as shown in the literature [19,32,36], and therefore the percentage of pores increased (Table 3). The presence of large Fe plate-like phases leads to formation of smaller eutectic Si particles, as shown in Figure 3c, and especially in Figure 3d. The finer eutectic Si particles could affect mechanical properties of the alloy because the presence of fine eutectic Si leads to increase of mechanical properties [37,38].

The metallography results demonstrated that the secondary alloys (B and C) had a higher amount of brittle Fe plate-like phases. Alloy B had the longest brittle Fe-rich plate-like phases, and in this case should have the lowest mechanical properties.

**Figure 4:**
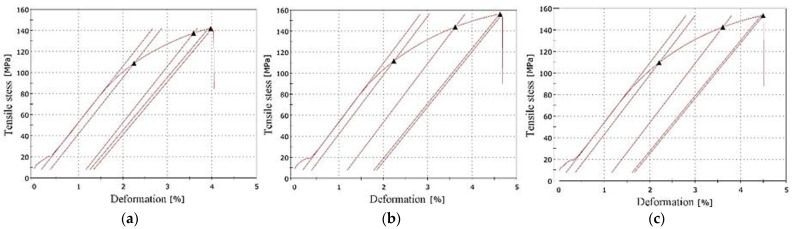
The tensile test diagrams of experimental alloys (**a**) A, (**b**) B and (**c**) C.

**Figure 5:**
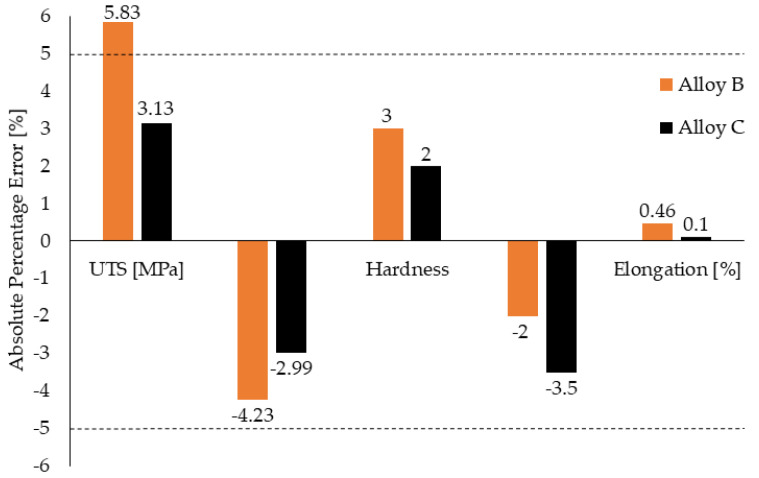
Mechanical properties evaluation of the absolute percentage errors of alloys B and C to alloy A (as received).

The presence of higher amount of Fe, higher content of Fe-rich phases, and porosity did not lead to a rapid decrease of mechanical properties (Figure 4, Table 4). Sacinti et al. [39] demonstrated that increasing shrinkage porosity caused by the growing of an amount of β-Al_5_FeSi, leads to a 3-fold decrease in the tensile elongation values. Moreover, the higher Fe content led to an increase in the mechanical properties (Figure 4), but the increase did not exceed the common measurement error of 7%, according to standard (in comparison to alloy A) (Figure 5). The increase in the mechanical properties of materials with the higher Fe content also decelerated [12]. This increase could correlate with the presence of the finer eutectic Si particles, as shows in Figure 3. The results also indicate that, even though Fe plate-like phases were formed, their effect on decreasing the mechanical properties was negligible. The results of the mechanical properties tests correlate with the results of the Fe_crit_ and Mn/Fe ratio tests (Table 2 and Table 4). Ultimate tensile strength (UTS), elongation A, and hardness (HBW) were higher in the alloys with the longer of Fe-rich plate-like phases (higher Fe_crit_). In contrast the highest YS was obtained for material with the chemical composition matching the standard (correlate with Mn/Fe ratio), andwhich was comparable with the results of Mathew et al. [12].

**Figure 6:**
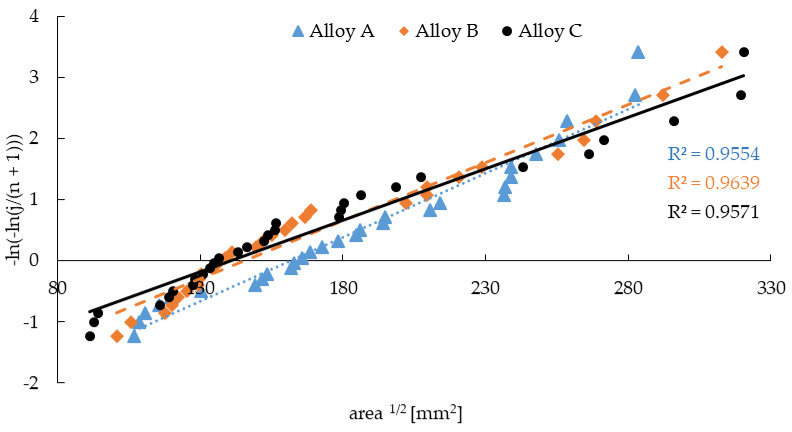
Results of the LEVD method.

The results of the quantitative analysis on the metallographic samples were used to predict the maximum defect size in all the specimens according to Murakami’s LEVD method (Table 5). This prediction was chosen for comparison with whether the real pores on the metallographic samples could be used to predict the size of porosity in the real cast or the experimental specimens with different dimensions (size), which would facilitate work in companies without CT or other non-destructive analyzers. The largest defect size measured on the experimental samples is presented as a Gumbel plot (Figure 6). The metallography samples had an area of 254 mm^2^ and the specimens for fatigue properties were 50 mm^2^, therefore the LEVD methods were used to predict the largest defect for these two areas and 1000 mm^2^ (Table 5).

The predicted defect size is more significant than the measured maximum defect size on the metallographic samples, and shows that the highest pores found were on specimens C. The Prediction of the largest defect size on a 50 mm^2^ area (corresponding with the area of the fatigue specimen) shows that alloys A and B had similar sizes of such defects, but alloy C had about a 6.3% greater defect size. Therefore, specimens A and B could have comparable fatigue properties, but the predicted area for the fatigue specimens (50 mm^2^) was smaller than measured (254 mm^2^), and according to Konečná et al. [29] and Murakami [31] these results may be distorted. It is also important to incorporate the effect of the large Fe-rich phases and their amount, because they were the largest most numerous in sample B (Table 3, Figure 3).

**Figure 7:**
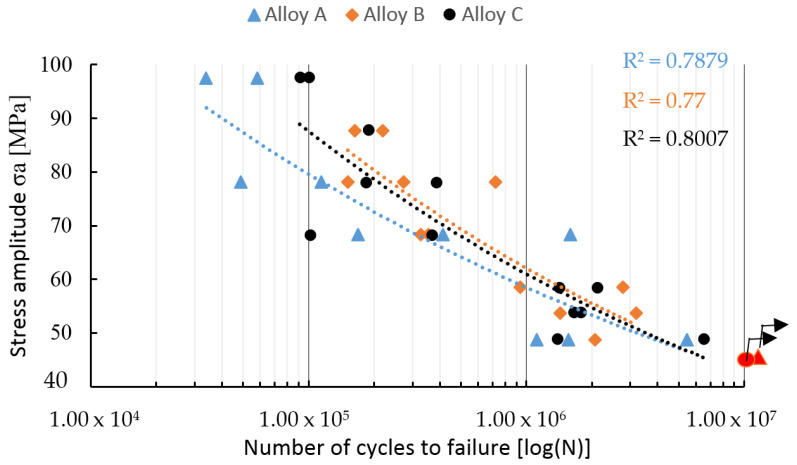
The S–N curves.

The fatigue properties results confirmed that specimens from the experimental alloys had different fatigue life at the same stress amplitude (Figure 7). These differences resulted from the presence of microstructural inhomogeneity, such as porosity or large Fe-rich or Si particles, as reported by Samuel and Samuel [40] and Dai et al. [41].

**Figure 8:**
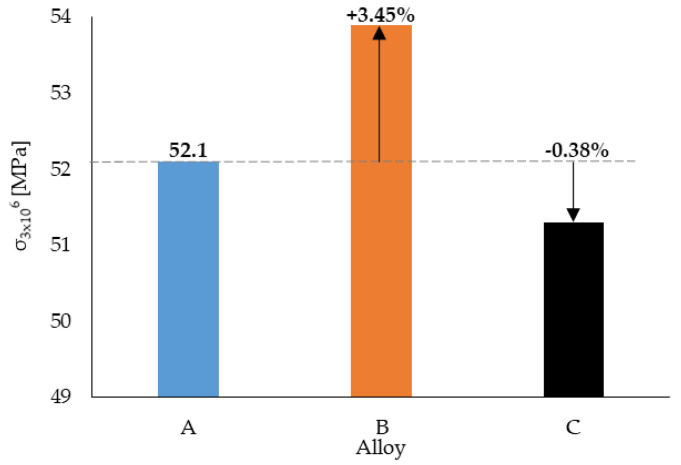
Evaluation of the fatigue life for the 3 × 10^6^ cycle alloys used in this study.

The samples that were not broken before reaching the specified number of cycles were defined as run-out and marked by red dots in Figure 7. The run-out was observed only for alloys A and C. This observation was probably related to results of the lengths of the Fe-rich phases and their quantity, because these phases are brittle and could act as concentrators of stress and stress risers for crack initiation [12,25,35,36,40,41]. However, the results shown in in Figure 7 reflect that the experimental alloys with higher Fe content had the better fatigue properties (alloy B being the best) under higher stress amplitude. The fatigue life setting for 3 × 10^6^ cycles shows that alloy B still had the highest fatigue life (Figure 8), and the fatigue life was established at about 52 MPa.

The possibility to create and use the Basquin Equations (5)–(7) for the prediction of fatigue life for different number of cycles [42] shows that the order is different under a lower stress amplitude:

Alloy A: σ_a_ = 372.02 × N_f_^(−0.134)^, (5)

Alloy B: σ_a_ = 567.35 × N_f_^(−0.160)^, (6)

Alloy C: σ_a_ = 535.69 × N_f_^(−0.157)^. (7)

As the experimental alloys were exposed to the stress amplitude of 40 MPa, the predicted number of cycles to failure have changed as follows: 1.689 × 10^7^ for alloy A, 1.580 × 10^7^ for alloy B, and 1.505 × 10^7^ for alloy C. The results confirmed that with a decrease of the stress amplitude, the fatigue properties of alloys with a higher content of Fe fell sharply, and were lowest in the alloys with chemical composition closest to the standard (alloy A).

**Figure 9:**
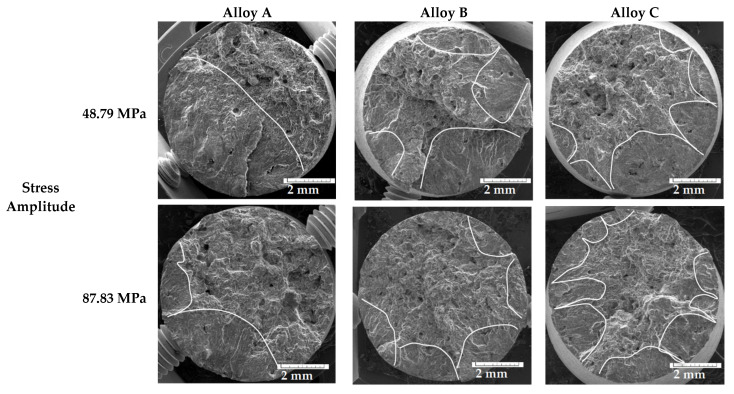
Fractography analysis of fatigue fracture under different stress amplitudes.

Fatigue life at higher-stress amplitude correlates with an order of experimental alloys according to Fe_crit_ (Table 2). Fatigue life under lower-stress amplitude correlates with an order of experimental alloys according to the Mn/Fe ratio (Table 2). Therefore, an increase in the fatigue life of the experimental alloys with the higher Fe content at higher-stress amplitude could correlate with the presence of Fe plate-like phases with length below 500 µm and with a higher surface ratio of pores, which led to the higher number of initiation places (Figure 9). The higher number of initiation places could lead to greater energy consumption for the propagation of fatigue fractures. The fatigue results and fractography analysis have not confirmed the results of Wang et al. [43] or Caceres and Griffiths [44] suggesting that large Fe-rich phase particles are the main reason for failure.

The predicted fatigue properties, according to the size of pores quantified by the LEVD, were also not confirmed. The fractography analysis (Figure 9) of the fatigue specimens also demonstrated the significant influence of a higher number of pores and Fe-rich plate-like phases on the formation of fatigue rupture. The lower-stress amplitude led to the formation of a larger fatigue region (delimited areas by white) and lowered the static rupture region (Figure 9). The specimens with the higher Fe content had many initiation sites, but with a lower fatigue region (Figure 9).

The differences of the fatigue results obtained from single experimental alloys under the same stress amplitudes were studied by CT analysis (Table 6). According to previous studies [19,23,25,32,33], specimens that were broken have a greater number and size of defects than the specimens which are not broken under the same stress amplitude. The results of the quantitative and statistical CT methods (Table 6) present the pore distribution along the active zone of the specimens and on the fracture surface (Figure 2). The length of the Fe-rich phases was measured on fatigued surfaces prepared by standard metallography methods using NIS Elements software.

**Figure 10:**
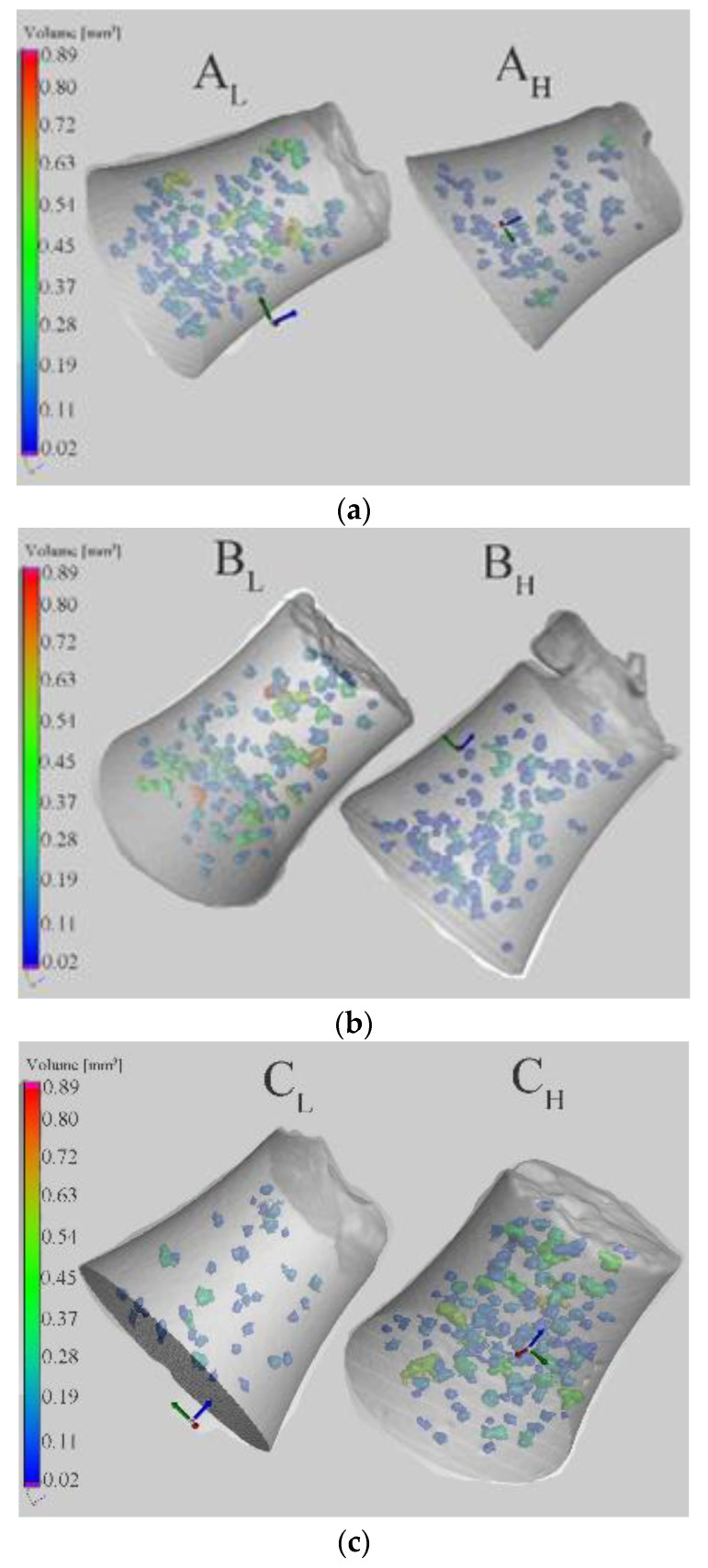
The CT analysis of porosity volume (mm^3^) in the active zone of (**a**) alloy A, (**b**) alloy B and (**c**) alloy C.

Alloys with subscript L represent materials with lower fatigue life, and alloys with subscript H represent materials with higher fatigue life. The results given in Table 6 show that the volume and ratio of porosity and the average length of the Fe needles were higher in the specimens with the lower fatigue life, which correlates with the results of previous studies [19,23,25,32,33]. The slight differences are in the specimen of alloy C (Table 6, Figure 10), where the porosity on the whole active zone of specimen C_L_ was about three times smaller than that of C_H_.

These results show that the length of the Fe-rich needles was significant to this case. Specimen C_L_ had Fe-rich phases about three times longer than those of C_H_. These results confirmed the Koutiri et al. [45] result that two different fatigue crack initiation mechanisms control fatigue damage in aluminum alloys: one mechanism is associated with relatively large micro-shrinkage pores, and the other is controlled by microstructural heterogeneities (Si particles, intermetallic phases) or the material matrix. The CT analysis results confirm that for the fatigue test the lengths of the Fe-rich phases were important, leading to rapidly decreasing porosity volume and ratio (Table 6) but causing the specimens to fracture earlier. However, the previous results confirm that experimental alloys with the higher length of the Fe plate-like phases had the smallest size of pores and therefore better properties. The length of Fe plate-like phases was important. The results demonstrate that if the Fe plate-like phases had a length of below 50 μm, the fatigue properties depended more on the porosity size. If the Fe needle length was greater than 50 μm, then the properties were mainly affected by the length of these Fe phases.

**Figure 11:**
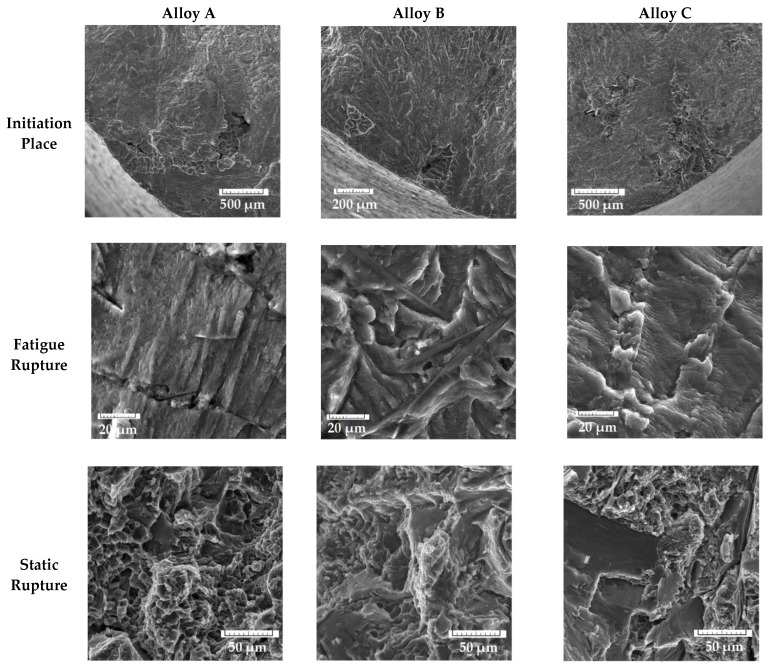
Fractography analysis of fatigue rupture at the same stress amplitude of 48.49 MPa.

As mentioned above, the initiation sites were sites of porosity on or near the surfaces (Figure 11). The presence of Fe-rich phases did not lead to initiation of fracture, but in all the experimental materials, fracture spreads come from pores.

The presence of the higher number of initiation places in specimens with higher Fe content correlates with the results of the porosity ratio (Table 3). The typical fatigue fracture of a matrix was observed on each specimen (Figure 11), but the presence of striation was difficult to observe, because of oxide films. The main micrography characteristics of the fatigue fractures near the initiating site were tear ridges (Figure 11) in the longitudinal direction to the crack propagation and fatigue striation in a direction perpendicular to the crack propagation. Differences were observed in the length of the fatigue fracture of the matrix since the specimens of alloy A have these areas larger compared to the other two alloys with higher Fe amount (Figure 11). This result was related to fineness of the microstructure (Figure 3b–d). Alloy B and C had long platelet-like phases in the fatigue regions. The final static rupture shows a mixed transcrystalline ductile and transcrystalline cleavage fracture. Transcrystalline ductile fractures relate to the matrix and the size of dimple depends on the fineness of the eutectic Si particles [11,35,46,47,48]. Transcrystalline cleavage fractures depend on the size and amount of Fe phases and eutectic Si particles. The results show that the cleavage fracture increased with increasing Fe quantities in the experimental alloys. These results correlate with results of previous studies [47,48].

**Figure 12:**
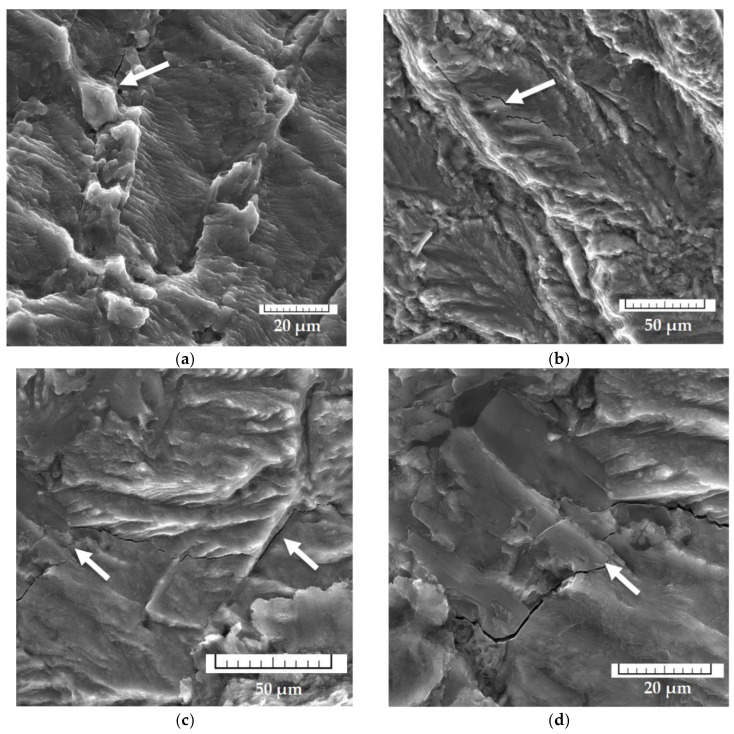
Propagation of the secondary cracks (**a**) in eutectic, (**b**) in the matrix, (**c**,**d**) along the Fe needles.

The fractography analysis also shows that the secondary cracks in the alloys with higher Fe quantities propagated along with the matrix at the eutectic region or through the matrix (Figure 12). When reaching the Fe platelet-like phases, they had to go around the phase and then propagate further (Figure 12), or sometimes we observed that the fractures stopped. These results confirm a crack climbs along the complex shape of Fe phases [49]. These results confirm the work of previous studies [12,25,35,36], showing that crack propagation occurs through the interface boundary of the particles and aluminum matrix.

**Figure 13:**
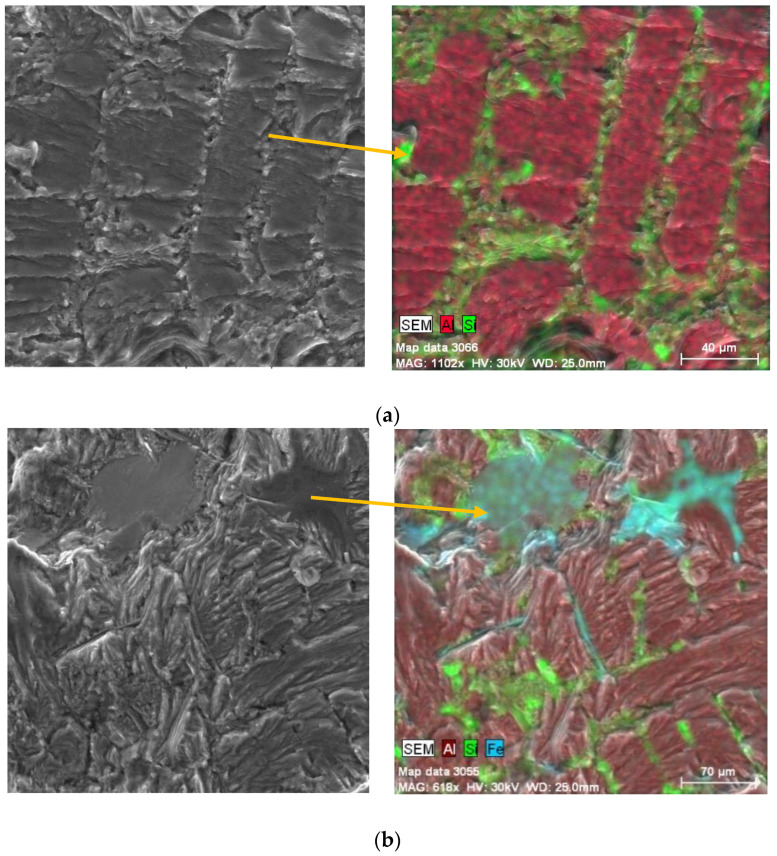

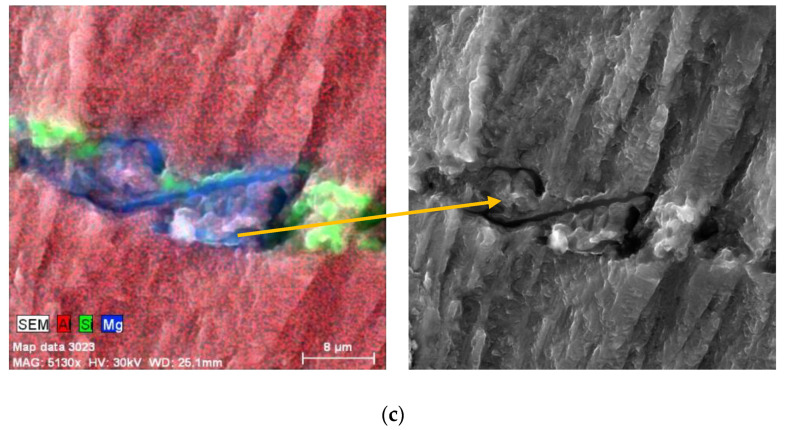
Mapping EDX analysis of (**a**) eutectic Si, (**b**) Fe plate-like phases, and (**c**) Mg-rich phases on fatigue fracture.

EDX analysis was used for declaration of the presence of phases in the fatigue fracture during the fractography assessment. As shown in Figure 13a, the eutectic silicon particles were observed, along with the dendritic structure of the matrix. The presence of the Fe needle phases depended on their orientation. They were observed in the form of thin needles, or long plates, in the fatigue fracture (Figure 13b). The morphology depended on the orientation of phases in the microstructure. The Mg phases (Mg_2_Si) were observed in the interdendritic region as eutectic (Figure 13c).

## 4. Conclusions

This study presents an analysis of the microstructure and property changes of AlSi_7_Mg_0.3_ alloys affected by higher Fe content. The benefits of this study are results for materials without Mn alloying and heat treatment, which are commonly used in production of aluminum cast products. The results of this study demonstrate the great effect (but not negative) of higher Fe content on β-Al_5_FeSi phase formation and property changes in AlSi_7_Mg_0.3_ cast alloys.

The new benefits of this study are:

- The non-usage the Mn addition or heat treatment does not affect the mechanical or fatigue properties of such experimental alloys too negatively.
- Gravity die sand mold casting does not lead to greater length of the Fe phases. The size of the Fe-rich plate-like phases was up to 500 µm, which corresponds to those of the secondary alloys cast under pressure into metallic molds. The size of the Fe-rich plate-like phases was up to 50 µm in experimental alloy A (the same chemical composition as primary alloys), despite the sand mold casting.
- The experimental alloy with the same chemical composition as primary alloys (A) does not have the enhanced formation of the Fe phases in needles form, despite the non-compliance with the Mn/Fe ratio guidelines.
- The highest Fe content (alloy C) did not lead to the longest needle phase formation, but these phases were thicker. This content led to increasing pore size and ratio. Therefore, the thickness of the phases correlates more with greater pore formation than with length.
- The length of the Fe needle-like phases affects the pore growth. The longest phases (in alloy B) led to formation of smaller pores than in alloys A and C.
- The formation of finer eutectic Si particles was confirmed as a result of the formation of the long and more Fe plate-like phases in the microstructure.
- The presence of a higher Fe content (also the Fe plate-like phases) does not lead to a decrease of the mechanical properties. The highest mechanical properties were found in specimen B with 0.454 wt.% of Fe, which had the longest Fe plate-like phases, but the difference was up to 7%, which is a common error range in mechanical tests, according to the standards.
- The results of the mechanical properties analyses showed that the order of experimental alloys, with regards to results of mechanical tests, correlates with the order according to the Fe_crit_ (for UTS, A and HBW) and Mn/Fe ratio (for YS). That means that the alloys with the highest value of Fe_crit_ or Mn/Fe ratio have the best properties.
- The results of the fatigue properties demonstrated that if the stress amplitude was higher, experimental alloy B (middle Fe content) had the best fatigue life. If the stress amplitude was lower, experimental alloy A (lower Fe content) had the best fatigue life.
- Moreover, the fatigue results show that the order (from the best to the worst) of experimental alloys correlates with results of Fe_crit_ if the stress amplitude is higher, and according to Mn/Fe ratio if the stress amplitude is lower.
- The CT analysis results confirmed the fact that if the Fe plate-like phases have a length of up to 50 µm, the fatigue properties depended more on the size of porosity. If the length of the Fe needles was more than 50 µm, then the properties were mainly affected by length of these Fe phases.

The results of this study (for alloy cast into a sand mold by gravity die casting) correlating with the common knowledge for material casted under the high pressure are:

- The non-usage of other technological influences (modification, refining, heat treatment, the addition of Mn) in sand mold casting led to formation of the higher amount of the Fe phases in the plate-like (needle) form and to an increase in the surface and volume of the pore ratio.
- The calculation of the Fe_crit_ and Mn/Fe ratios were successfully used for the prediction of plate-like (needles) Fe-rich phase formation. The critical level of Fe was reached in the experimental alloys with the secondary composition (B and C). In addition, the Mn/Fe ratio was not lower than 0.5 for these experimental materials.
- Increasing the Fe content leads to the formation of thicker Fe plate-like phases, which are connected to each other and create a difficult shape.
- The study also shows that use of Murakami’s LEVD method used for prediction of the maximum defect size was not sufficient for any of the specimens, and it was not relevant for the prediction of fatigue properties or the maximum defect size in our experimental alloys.
- The fractography analysis of fracture surfaces shows that the surface consisted of initiation place, fatigue fracture propagation, and final static rupture. The initiation places were the pores in all cases. The typical fatigue fracture with striation was also observed, but the striations were observed sporadically due to the presence of oxide films. The final static rupture consisted of transcrystalline ductile (matrix) and transcrystalline cleavage fractures (Fe-rich phases and Si particles). The assessment of the fatigue fracture showed that fatigue properties could depend on the higher number of initiation places. It seems that at the higher stress amplitude, initiation of the fracture came from pores. However, if the crack reached the long Fe plate-like phases, the propagation stopped and the new places for growth were created, or the crack climbed along with the difficult shape of the Fe-rich phases.

## Figures and Tables

**Table 1:** Chemical composition of experimental alloys, in wt.% (remainder Al).

Alloy	Si	Mg	Fe	Mn	Ti	Zn	Cu	Sn	Na	Sr
A	7.028	0.354	**0.123**	0.009	0.123	0.036	0.013	0.004	0.002	<0.001
B	7.340	0.302	**0.454**	0.009	0.128	0.020	0.021	0.006	0.004	<0.001
C	7.315	0.292	**0.655**	0.010	0.120	0.028	0.030	0.005	0.005	<0.001

**Table 2:** The critical Fe levels and ratio of Mn/Fe for experimental alloys.

Alloy	A	B	C
Fe_crit_	0.4771	0.5005	0.4986
Mn/Fe	0.0732	0.0198	0.0153

**Table 3:** Results of quantitative analysis with NIS Elements on metallographic samples.

Alloy	Quantification of Porosity						Quantification of β-Fe Phases		
	Pore Surface Size (µm^2^)			Pore Surface Ratio (%)			Length of β-Fe Phases (µm)		
	Min	Max	Average	Min	Max	Average	Min	Max	Average
A	775	130,423	20,208	0.5	3.9	1.6	10.49	52.47	30.04
B	674	97,890	18,937	0.6	4.2	1.8	12.06	202.46	59.77
C	1342	104,040	22,833	1.2	3.5	2.3	8.29	198.65	52.57

**Table 4:** Mmechanical properties of experimental alloys.

Alloy	UTS (MPa)			YS (MPa)			A (%)			HBW 5/250/15		
	Min	Max	Average	Min	Max	Average	Min	Max	Average	Min	Max	Average
A	132.26	148.61	140.9	83.26	110.58	100.91	1.18	1.68	1.45	50.5	55.8	52
B	139.54	156.12	150.22	81.74	111.62	96.68	1.69	2.13	1.91	52.8	57.9	55
C	134.95	153.41	147.05	85.92	109.91	97.915	1.5	1.66	1.55	51	56.8	54

**Table 5:** Prediction of the largest defects.

Alloys	The Square Root of the Measured Maximum Defect Size on 254 mm^2^ with Quantitative Analysis (mm^2^)	Predicted Largest Defect Size on 50 mm^2^ with Using LEVD (mm^2^)	Predicted Largest Defect Size on 254 mm^2^ with Using LEVD (mm^2^)	Predicted Largest Defect Size on 1000 mm^2^ with Using LEVD (mm^2^)	Differences in Measured and Predicted Defect Size (%)
A	361.1412	318.0962	396.5847	462.2802	9.6
B	312.8738	318.0961	404.7144	477.2575	22.6
C	322.5523	338.3548	434.6887	515.3211	25.6

**Table 6:** CT porosity analysis results, quantitative analysis of the Fe-needle phases and fatigue properties.

Alloy *	A_L_	A_H_	B_L_	B_H_	C_L_	C_H_
**Fatigue tests results**	**-**					
stress amplitude (MPa)	68.31	68.31	53.79	53.79	68.31	68.31
number of cycles to failure	169,815	1,575,900	1,430,000	3,190,000	102,000	370,000
**CT analysis results**	**-**					
ratio of porosity along active zone (%)	2.38	1.32	2.55	1.75	0.66	2.07
volume of porosity on fatigue surface (mm^3^)	0.117	0.110	0.145	0.125	0.120	0.113
volume of porosity along active zone (mm^3^)	18.46	9.12	20.36	14.65	4.92	16.33
materials volume (mm^3^)	756.24	681.13	778.08	818.94	735.81	771.99
average length of β-Fe (µm)	43.03	27.56	44.62	28.16	170.97	59.21

* L—Lower Fatigue Life, H—Higher Fatigue Life.

## Data Availability

Data sharing not applicable.
